# Identification of methylation signatures associated with CAR T cell in B-cell acute lymphoblastic leukemia and non-hodgkin’s lymphoma

**DOI:** 10.3389/fonc.2022.976262

**Published:** 2022-08-11

**Authors:** Jiwei Song, FeiMing Huang, Lei Chen, KaiYan Feng, Fangfang Jian, Tao Huang, Yu-Dong Cai

**Affiliations:** ^1^ College of Life Science, Changchun Sci-Tech University, Shuangyang, China; ^2^ School of Life Sciences, Shanghai University, Shanghai, China; ^3^ College of Information Engineering, Shanghai Maritime University, Shanghai, China; ^4^ Department of Computer Science, Guangdong AIB Polytechnic College, Guangzhou, China; ^5^ Ruijin Hospital, Shanghai Jiao Tong University School of Medicine, Shanghai, China; ^6^ Bio-Med Big Data Center, CAS Key Laboratory of Computational Biology, Shanghai Institute of Nutrition and Health, University of Chinese Academy of Sciences, Chinese Academy of Sciences, Shanghai, China; ^7^ CAS Key Laboratory of Tissue Microenvironment and Tumor, Shanghai Institute of Nutrition and Health, University of Chinese Academy of Sciences, Chinese Academy of Sciences, Shanghai, China

**Keywords:** CAR T cell, B-cell acute lymphocytic leukemia, B-cell acute non-Hodgkin’s lymphoma, feature selection, classification algorithm, classification rule

## Abstract

CD19-targeted CAR T cell immunotherapy has exceptional efficacy for the treatment of B-cell malignancies. B-cell acute lymphocytic leukemia and non-Hodgkin’s lymphoma are two common B-cell malignancies with high recurrence rate and are refractory to cure. Although CAR T-cell immunotherapy overcomes the limitations of conventional treatments for such malignancies, failure of treatment and tumor recurrence remain common. In this study, we searched for important methylation signatures to differentiate CAR-transduced and untransduced T cells from patients with acute lymphoblastic leukemia and non-Hodgkin’s lymphoma. First, we used three feature ranking methods, namely, Monte Carlo feature selection, light gradient boosting machine, and least absolute shrinkage and selection operator, to rank all methylation features in order of their importance. Then, the incremental feature selection method was adopted to construct efficient classifiers and filter the optimal feature subsets. Some important methylated genes, namely, *SERPINB6*, *ANK1*, *PDCD5*, *DAPK2*, and *DNAJB6*, were identified. Furthermore, the classification rules for distinguishing different classes were established, which can precisely describe the role of methylation features in the classification. Overall, we applied advanced machine learning approaches to the high-throughput data, investigating the mechanism of CAR T cells to establish the theoretical foundation for modifying CAR T cells.

## Introduction

The chimeric antigen receptor (CAR) T cell immunotherapy is a type of pericyte therapy in which T cells are genetically modified to express chimeric antigen receptors that detect and kill tumor cells in patients (1). In the USA, over 70,000 people are diagnosed with non-Hodgkin’s lymphoma (NHL) annually, with a 5-year survival rate of roughly 70% (2). Acute lymphoblastic leukemia (ALL) is the most common pediatric cancer, accounting for approximately 25% of all pediatric cancer cases, and has a high recurrence rate (3). CD19-targeted CAR T-cell immunotherapy has a high response rate in B-cell ALL and B-cell NHL, especially in ALL, with a treatment effectiveness of 90% (4). However, CAR-T therapy is not effective for all tumor patients, and drug-resistant relapse occurs in approximately 50% of patients treated with CD19-targeted CAR-T (5). Some CAR T cells become exhausted, resulting in an increase in inhibitory receptors and a loss of effector function (6, 7). Creating a long-lasting therapeutic response is an essential problem that demands a better knowledge of the cellular and molecular processes that drive CAR T cell proliferation, contraction, and persistence in patients. Studying the specific functions of CAR-transduced T-cells at the molecular level, such as epigenetic level, can help in the understanding of the deeper mechanisms of CAR-T cell immunotherapy and clinical identification of potential targets for effective cancer treatment.

CAR consists of an antigen recognition domain, a co-stimulatory region, and a T cell activation region (8–10). Through multiple signaling cascades, the costimulatory region and T cell activation region activate the CAR T cells, which exhibit proliferative and cytotoxic properties (11). Activated CAR T cells have different gene expression patterns compared with regular T cells, which are influenced by epigenetic modifications (12). DNA may be modified in various ways, the most frequent of which is direct nucleotide methylation. Methylation of promoters results in a decrease in gene expression and suppression of transcription. High-expression genes have high levels of methylation at introns but low levels of methylation at the promoter or regulatory areas (13, 14). Epigenetic imprinting is emerging as a unifying subject in the study of immunological memory and the correlation of long-lasting antitumor responses.

Modifications in DNA methylation shape the overall immune response by altering the phenotype and function of CAR T cells. Zebley et al. showed that alterations in DNA methylation are linked to the proliferation and contraction of CAR T cells and that CD19-targeted CAR T cells acquire DNA methylation features over time. These results suggested that these cells are developing into a progenitor subset of exhausted T cells (15). Meanwhile, Wang et al. discovered that CAR T cells treated with low doses of the demethylating drug decitabine had stronger antitumor, proliferation, and cytokine release abilities. This result indicates the presence of methylation in CAR T cells that inhibit their oncogenic functions (16). Among the large number of methylation sites, traditional biological experiments cannot meet the requirement of searching for methylation sites that affect the proliferation, failure, and oncogenic functions of CAR T cells. Therefore, this study was focused on how to combine advanced computational methods, such as machine learning, to mine CAR T cell-specific methylation sites to find potential sites for the sustained activation of CAR T cells.

Herein, we devised a process to rapidly screen CAR T cells for specific methylation sites. First, the methylation sites were analyzed and sorted by three feature ranking methods, namely, least absolute shrinkage and selection operator (LASSO) (17), light gradient boosting machine (LightGBM) (18), and Monte Carlo feature selection (MCFS) (19). Then, the incremental feature selection (IFS) (20) method was used to estimate the importance of feature subsets, which were constructed from three ranked methylation site lists, by evaluating the performance of classifiers on these subsets. One optimal feature subset was obtained from each list generated by one feature ranking method. The intersection of all obtained optimal feature subsets was investigated. The methylation sites that recurred multiple times were considered to be highly correlated with the specific functions of the CAR T cell, because the three feature ranking methods used different and independent concepts. Moreover, we also used decision trees (DTs) (21) to create quantitative classification rules that can accurately describe the composition of features for distinguishing each class. All in all, we identified the methylation sites associated with specific functions of the CAR T cells on a large scale using an efficient machine learning based framework and provided a functional description of highly ranked methylation sites in conjunction with the literature.

## Materials and methods

### Data and preprocessing

The T-cell methylation profiles of 157 patients with B-cell malignancies, including ALL and NHL, were downloaded from the GEO database under the accession number GSE179414 (22). The dataset comprised 77 ALL and 37 NHL cases, who were treated with CART19 cells. These two groups of patients were injected with CAR-transduced T cells and were referred to as ALL transduced and NHL transduced samples, respectively. Meanwhile, 13 ALL and 30 NHL cases were also included in the dataset, but they were not given CART19 cells. These patients were injected with CAR-untransduced T cells and were referred to as ALL untransduced and NHL untransduced samples. Each group was deemed as a class in this study. We investigated their essential differences by studying the classification problem on these classes. Furthermore, each sample in the dataset was represented by 865,859 methylation sites. These sites were termed as features in this investigation.

### Feature ranking methods

A large number of methylation sites were involved in the investigated methylation profiles, which were deemed as features in this study. Evidently, a small proportion of features were highly related to distinguish the CAR-untransduced and -transduced T cells. The powerful feature analysis method in machine learning was necessary. Here, three such methods were employed, including MCFS (19), LightGBM (18), and LASSO (17).

#### Monte Carlo feature selection

The MCFS algorithm is a DT-based method for determining the relevance of features. This method was first proposed by Micha et al. and has been widely used in tackling various complex medical and biological problems, showing promise in solving such problems (19, 23, 24).

The procedures of MCFS can be summarized as the following steps: (1) *s* feature subsets are randomly constructed from all features; (2) For each feature subset, *t* DTs are constructed by randomly selecting training and test samples from the original datasets; (3) After t×s DTs have been built, each feature *g* is evaluated by the relative importance (RI), which can be computed as follows:


(1) $$RI_{g}=\;\sum_{\tau =1}^{st}{\left(wAcc\right)}^{u}\sum_{n_{g}\left(\tau \right)}IG\left(n_{g}\left(\tau \right)\right){\left(\frac{no.in\;n_{g}\left(\tau \right)}{no.in\;\tau }\right)}^{v},$$

where wAcc is the weighted accuracy; IG(ng(τ)) stands for the information gain (IG) of ng(τ) (a DT node with the feature g); no.in ng(τ) stands for the number of samples in ng(τ); no.in τ stands for the sample sizes in the tree root; and u and v are two settled positive integers. According to the RI values of all features, they are ranked in a feature list by the decreasing order of their RI values.

In this study, we adopted the MCFS program downloaded from http://www.ipipan.eu/staff/m.draminski/mcfs.html. Default parameters were used to execute such program, where *u* and *v* were set to one.

#### Light gradient boosting machine

LightGBM is an iterative boosting tree classifier proposed by Microsoft and is a modified version of the gradient boosting DT (18). LightGBM uses the total number of times (i.e., T_Split) that each feature is involved in the trees iteratively created and the gain (i.e., T_Gain) that a feature is utilized for splitting in all DTs as measurements of feature relevance for the prediction. They are defined as


(2) $$T\_Split=\;\sum_{t=1}^{K}Split_{t},$$

(3) $$T\_Gain=\;\sum_{t=1}^{K}Gain_{t},$$

where K is the K DTs generated by K iterations. Here, we used T_Split as a metric to measure the importance of features, i.e., features were sorted in the decreasing order of their T_Split values.

This study adopted the LightGBM program retrieved from https://lightgbm.readthedocs.io/en/latest/. It was performed with its default parameters.

#### Least absolute shrinkage and selection operator

The LASSO algorithm is a feature selection method based on linear regression models that selects and compresses variables to prevent overfitting (17). This method uses the L1 paradigm to create a penalty function that selectively removes lower-correlation variables by imposing a bigger penalty on the larger value of the feature variables. This process results in a model with fewer feature variables and effectively avoiding overfitting. If the coefficients of the input features did not contribute positively to the training of the machine learning model, they were scaled down. As a result, the features could be ranked according to their coefficients.

Here, the LASSO package integrated in Scikit-learn (25) was used and its default parameters were adopted.

### Incremental feature selection

By three feature ranking methods, all features were ranked in three lists. Evidently, top features in each list were important. However, determining the number of top features was still a problem. Thus, the IFS method (20, 26–28) was employed, which can determine the suitable number of top features. The procedures of IFS method can be divided as follows: (1) Several feature subsets are constructed based on the ranked feature list, which consists of some top features in the list; (2) A classifier is constructed on samples represented by features in each subset and its performance is evaluated by ten-fold cross-validation (29); (3) The classifier with the best performance can be found and the feature subset used in this classifier is picked up as the optimal feature subset. As three ranked feature list was produced in this study, IFS method was executed on each list. Three optimal feature subsets were obtained. We drew Venn diagrams for these three feature sets to display and analyze their intersection results.

### Synthetic minority oversampling technique

As described in Section Data and preprocessing, the size of the class with the most samples (77) was about six times as large as that of the class with the least samples (13). Given the imbalance in sample size, when building and evaluating the classifiers, the predicted results would be biased toward the classes with a larger sample size, reducing the generalization ability for the model. In view of this, synthetic minority oversampling technique (SMOTE) algorithm was used in this study to effectively achieve data balance by enlarging the size of each minority class (30, 31). It generates new samples for each minority class by the linear combination of two samples in the same minority class, which are near enough. Finally, all classes have the same number of samples. This study adopted the SMOTE program obtained from https://github.com/scikitlearn-contrib/imbalanced-learn. Likewise, its default parameters were used.

### Classification algorithm

In the IFS method, classifiers were built to evaluate the importance of constructed feature subsets. A certain classification algorithm was necessary to execute the IFS method. In this study, we applied four classification algorithms, namely, K-nearest neighbor (KNN) (32), support vector machine (SVM) (33), random forest (RF) (34), and DT (21). The purpose of employing these algorithms was to fully test the importance of each constructed feature subset and select the best one.

The KNN algorithm is one of the most classic classification algorithms. Its principle is quite simple. However, its performance is still acceptable in some cases. Given a test sample, KNN finds its *k* nearest neighbors in the training dataset. According to the labels of these neighbors, the label of the test sample can be determined.

The SVM is a classification algorithm based on statistical learning theory. It generally maps samples into a high-dimensional space by using a kernel function and linearly separates them by finding the maximum margin separating hyperplane. For a test sample, it is also mapped into the same high-dimensional space and its class is determined by the side of hyperplane that the test sample is located.

The RF is also a classic classification algorithm, which is quite different from SVM. In fact, it is an ensemble algorithm, which contains several DTs. Each DT is built by randomly selecting features and samples. For a test sample, each DT gives its prediction. RF integrates these predictions using majority voting.

For the above three classification algorithms, their classification principles are quite difficult for us to understand. Thus, few insights can be extracted from them. DT has its merits in this regard. It is a white-box algorithm, whose classification procedures are completely open, giving opportunities for us to uncover its principle learned from the given dataset, thereby access more knowledge from the dataset. A DT is a tree structure consisting of a series of nodes and branches that use logical operations. Two types of nodes are contained in a DT, they are branch and leaf nodes. The branch node is always related to one feature. According the threshold, samples in a branch node are classified into two groups. The leaf node stands for one class. Samples that reach such node are assigned the corresponding class label. During predictions, it starts at the root node and sorts the test samples down the tree according to the thresholds defined at each branch node. Furthermore, a DT can be represented by lots of if-then rules. Each rule is constructed by a path from the root node to one leaf node. From these rules, a clearer picture on each class can be uncovered.

Above algorithms have been applied to construct various classifiers in dealing with biological and medical problems (35–40). In this study, we used the corresponding Python Scikit-learn packages (25) of above four classification algorithms to implement them.

### Performance evaluation

To evaluate the performance of all classifiers constructed in the IFS method, several measurements were employed. First, as multi-class classifiers, the overall accuracy (ACC) was adopted, which is the most accepted measurements. It is defined as the proportion of correctly predicted samples among all samples. However, such measurement is not perfect if the sizes of classes are quite different. Thus, we also employed the Matthew correlation coefficients (MCC) (41), which is deemed as a balanced measurement. As four classes were involved, the MCC in multi-class was adopted, which is defined as


(4) $$MCC=\frac{cov\left(X,Y\right)}{\sqrt{cov\left(X,X\right)cov\left(Y,Y\right)}},$$

where *X* and *Y* are two binary matrices, indicating the true and predicted class of each sample.

In addition, we computed the precision, recall and F1-score for each class, which is defined as


(5) $$Precision=\frac{TP}{TP+FP},$$

(6) $$Recall=\frac{TP}{TP+FN},$$

(7) $$F1-score=\frac{2\times Precision\times Recall}{Precision+Recall}$$

where *TP* stands for the number of samples in such class which are correctly predicted, *FP* is the number of samples in other classes which are classified into this class, *FN* is the number of samples in such class which are wrongly predicted. According to F1-score on each class, the macro F1 and weighted F1 are further computed to give a whole evaluation on classifiers. For macro F1, it is defined as the mean of all F1-score values on all classes, whereas weighted F1 integrates all F1-score values by further considering the class sizes, that is, it is the weighted mean of F1-score values.

In this study, weighted F1 was picked up as the key measurement. Other measurements were provided as reference.

### Functional enrichment analysis

By analyzing the T-cell methylation profiles downloaded from the GEO with several machine learning algorithms, the optimal feature subsets, containing several methylation probes, were obtained. After taking the union operation on these subsets and mapping features in the union set onto the genes, ClusterProfiler in R was used to calculate the enrichment of these genes on GO terms and KEGG pathways (42). The p-value was corrected with FDR, and 0.05 was chosen as the cutoff value. Only the GO terms and KEGG pathways with FDR<0.05 were considered statistically significant.

## Results

**Figure 1:**
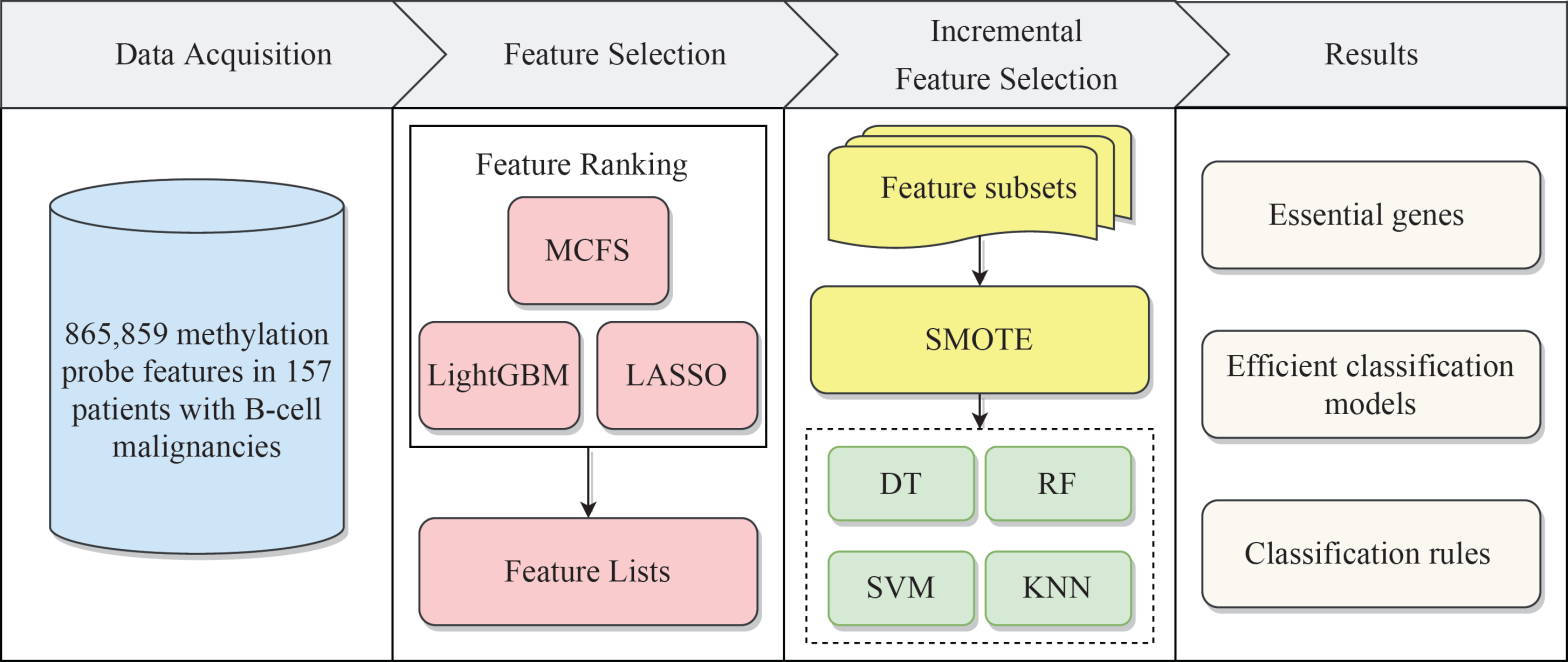
Flow chart of the entire analytical process. The 865,859 methylation probe signals on patients with B-cell malignancies were ranked according to feature importance by using three feature ranking algorithms, namely, MCFS, LightGBM, and LASSO. Then, three ordered feature lists were fed into the incremental feature selection (IFS) method, which incorporates four classification algorithms. Finally, based on the IFS results, the essential genes, efficient classification models and classification rules were extracted.

We built a machine learning based framework for analyzing CAR-transduced and untransduced T cells in different B-cell malignancies and further constructed efficient classifiers to discriminate CAR-transduced and untransduced T cells. The entire procedures are illustrated in **Figure 1**. The detailed results were listed in this section.

### Results of feature selecting methods

Each sample was represented by a large number of features (methylation sites). They were deeply analyzed by three feature ranking methods (MCFS, LightGBM, and LASSO). Each method produced one feature list, which is provided in **Table S1**. It was necessary to pointed out that only features with evaluation score (RI for MCFS, T_Split for LightGBM and coefficient for LASSO) larger than zero were provided in **Table S1**. The top-ranked features are considered to be important because of their participation in the classification. Their biological significance and the reasons why they are important as core classification features would be discussed in Section Discussion.

### Results of IFS method

The three ordered feature lists created by three feature ranking methods were fed into the IFS method one by one and four classification algorithms (DT, KNN, RF and SVM) were used in the IFS method. To save time, we only considered the top 1000 features in each list. For each list, 1000 possible feature subsets were constructed, on which 1000 classifiers with one give classification algorithm were built and evaluated by ten-fold cross-validation. The evaluated results, including measurements listed in Section Performance evaluation, are available in **Table S2**.

**Figure 2:**
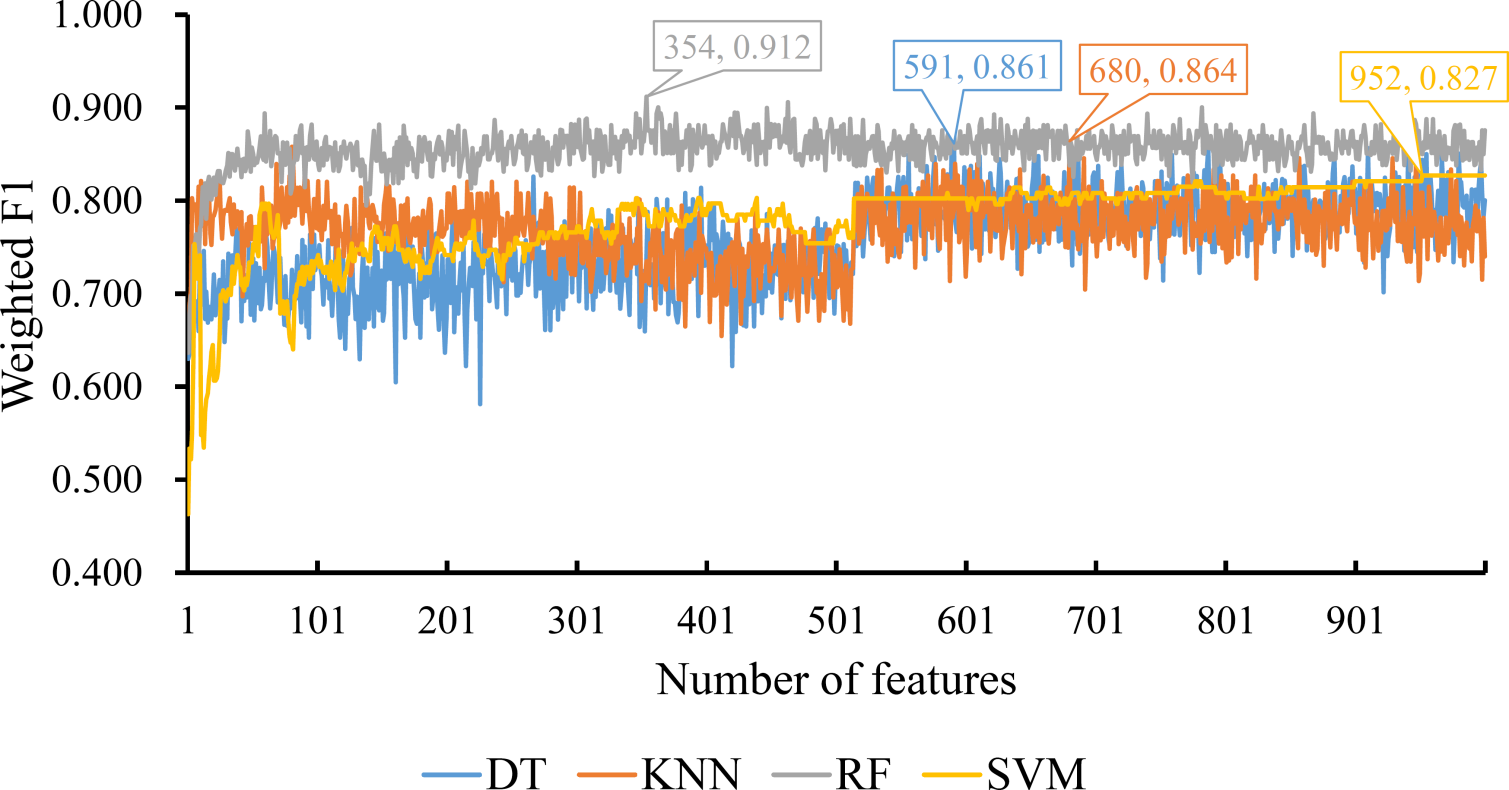
IFS curves for displaying the performance of four classification algorithms on the feature list yielded by MCFS method. The best classifiers on different algorithms yield the weight F1 values of 0.861, 0.864, 0.912 and 0.827, respectively, which use top 591, 680, 354 and 952, respectively, features in the list.

**Figure 3:**
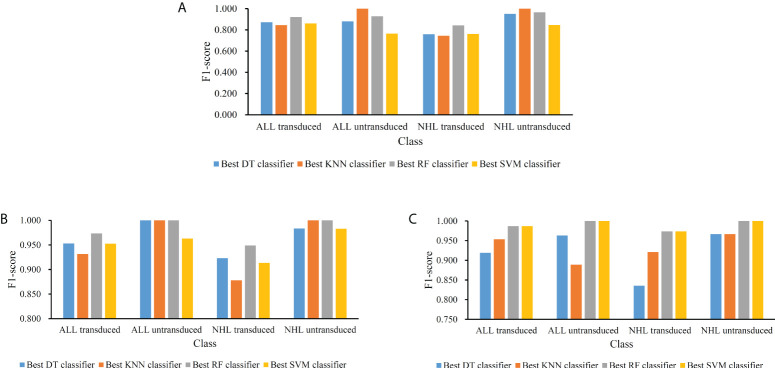
Performance of the best classifiers using different classification algorithms and feature lists on four classes. **(A)** Feature list generated by MCFS method; **(B)** Feature list generated by LightGBM method; **(C)** Feature list generated by LASSO method.

For the feature list yielded by MCFS method, we plotted an IFS curve for each classification algorithm to illustrate its performance on different feature subsets, which is shown in **Figure 2**. It can be observed that DT, KNN, RF and SVM can yielded the highest weighted F1 values of 0.861, 0.864, 0.912 and 0.827, respectively. These values were obtained by using top 591, 680, 354 and 952, respectively, features in the list, which comprised the optimal feature subsets for these four classification algorithms. With the optimal feature subsets, we can build the best DT, KNN, RF and SVM classifiers. The values of macro F1, ACC and MCC of these classifiers are listed in **Table 1**. Furthermore, their performance on four classes is illustrated in **Figure 3**. Evidently, the best RF classifier was superior to other three best classifiers and the best DT classifier was only better than the best SVM classifier.

**Table 1:** Performance of the best classifiers using different classification algorithms and feature ranking methods.

Feature ranking method	Classification algorithm	Weighted F1	Macro F1	ACC	MCC
MCFS	DT	0.861	0.866	0.860	0.792
	KNN	0.864	0.897	0.860	0.801
	RF	0.912	0.914	0.911	0.866
	SVM	0.827	0.808	0.822	0.746
LightGBM	DT	0.956	0.965	0.955	0.935
	KNN	0.938	0.952	0.936	0.909
	RF	0.975	0.981	0.975	0.963
	SVM	0.950	0.953	0.949	0.927
LASSO	DT	0.912	0.921	0.911	0.869
	KNN	0.943	0.933	0.943	0.914
	RF	0.987	0.990	0.987	0.981
	SVM	0.987	0.990	0.987	0.981

**Figure 4:**
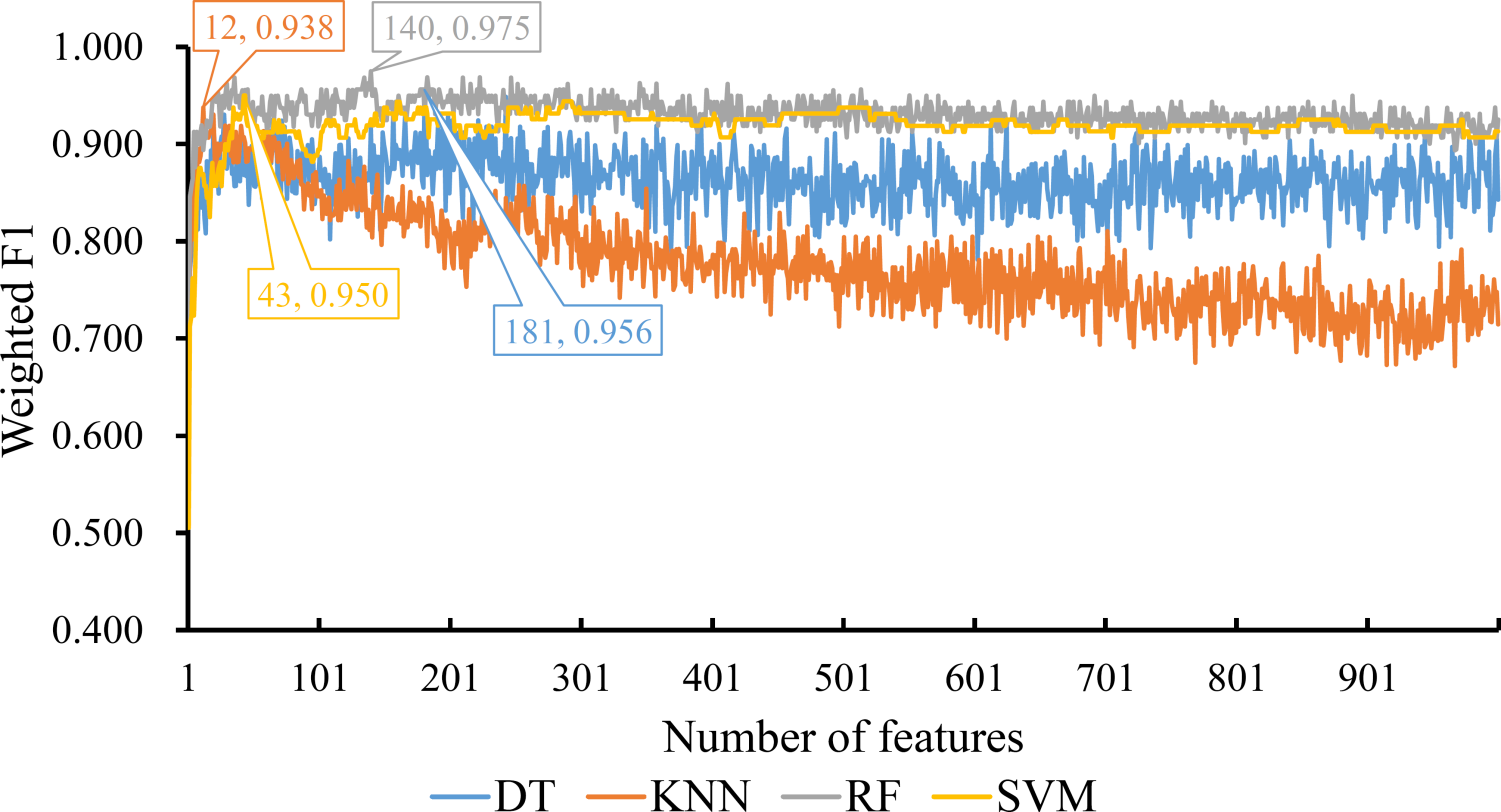
IFS curves for displaying the performance of four classification algorithms on the feature list yielded by LighGBM method. The best classifiers on different algorithms yield the weight F1 values of 0.956, 0.938, 0.975 and 0.950, respectively, which use top 181, 12, 140 and 43, respectively, features in the list.

For the feature list generated by LightGBM method, four IFS curves were also drawn, which are shown in **Figure 4**. When top 181, 12, 140 and 43 features in the list were adopted, four classification algorithms produced the highest weighted F1 values of 0.956, 0.938, 0.975 and 0.950, respectively. These features constituted the optimal feature subset for each classification algorithm. Furthermore, the best DT/KNN/RF/SVM classifier was built with its corresponding optimal feature subset. The detailed performance of these best classifiers is listed in **Table 1** and shown in **Figure 3**. Likewise, the best RF classifier still provided the highest performance. As for the best DT classifier, it was a little better than the best KNN and SVM classifiers.

**Figure 5:**
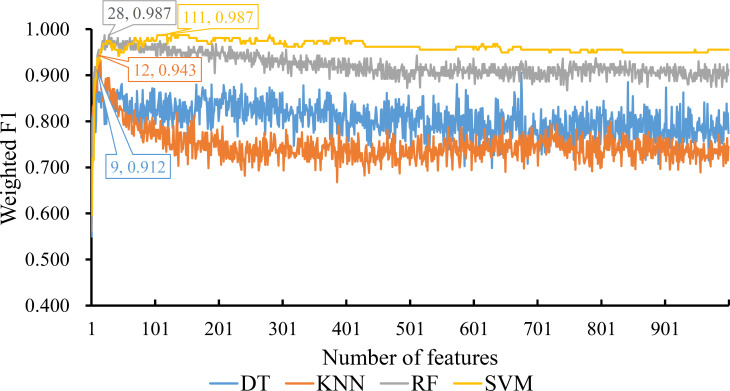
IFS curves for displaying the performance of four classification algorithms on the feature list yielded by LASSO method. The best classifiers on different algorithms yield the weight F1 values of 0.912, 0.943, 0.987 and 0.987, respectively, which use top 9, 12, 28 and 111, respectively, features in the list.

For the last feature list generated by LASSO method, IFS curves were also plotted, as shown in **Figure 5**. The highest weighted F1 for DT, KNN, RF and SVM were 0.912, 0.943, 0.987 and 0.987, respectively. To reach such performance, top 9, 12, 28 and 111, respectively, features were used. These features comprised the optimal feature subset for each classification algorithm. Similarly, the best DT, KNN, RF and SVM classifiers were constructed with the corresponding optimal feature subsets. **Table 1** and **Figure 3** provide their detailed performance. The best RF classifier provided equal performance to the best SVM classifier. However, the best RF classifier adopted much less features than the best SVM classifier. Thus, this classifier was still deemed to be better than other three classifiers. On the other hand, the best DT classifier gave the lowest performance among all four best classifiers.

Based on the above arguments, the best RF classifier always provided better performance than other three best classifiers on each feature list. Among three RF classifiers built on three feature lists, the RF classifier on the list generated by LASSO provided the highest performance. Such classifier can be a useful tool to discriminate CAR-transduced and untransduced T cells. On the other hand, the DT classifiers generally gave the low performance. However, they can provide more clues to uncover the differences between CAR-transduced and untransduced T cells.

### Feature intersection

As mentioned above, on each feature list yielded by one feature ranking method, the best RF classifier was always better than other three best classifiers. Thus, its optimal feature subset was picked up as the optimal feature subset for one feature ranking method. In detail, the optimal feature subsets for MCFS, LightGBM and Lasso consisted of the top 354, 140, and 28 features in the lists generated by MCFS, LightGBM, and LASSO, respectively.

**Figure 6:**
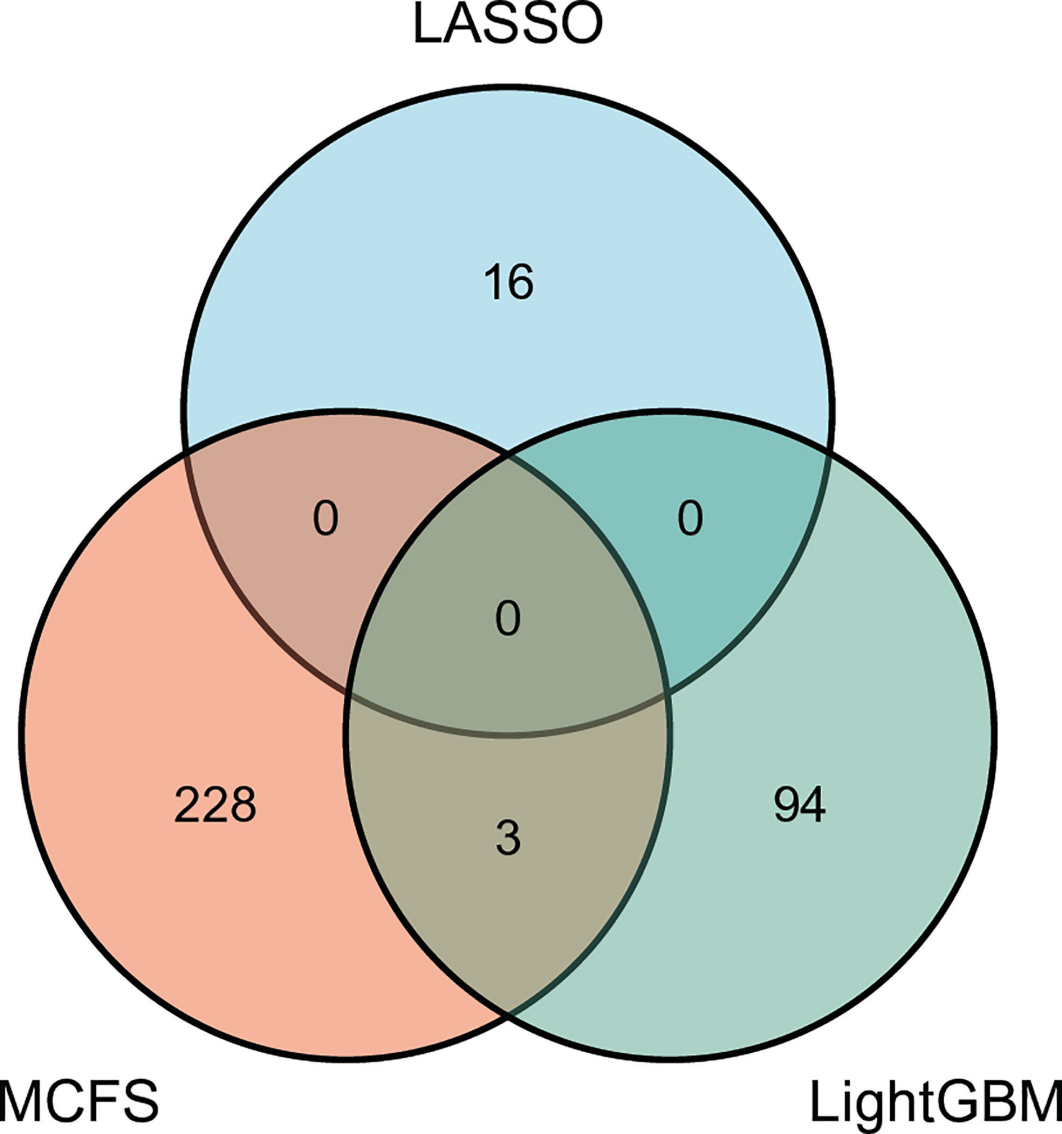
Venn diagram to show the intersection of the optimal gene sets for MCFS, LightGBM, and LASSO. Three genes are contained in two optimal gene sets, indicating their importance.

For each above-mentioned optimal feature subset, features in such subset were mapped onto their related genes, which comprised the optimal gene subset. Concretely, the optimal gene set for MCFS, LightGBM and LASSO contained 231, 97 and 16 genes, respectively. Detailed genes in these sets are provided in **Table S3**. The intersection of these three gene sets was investigated and plotted in one Venn diagram, as shown in **Figure 6**. It can be observed that no genes were contained in all three optimal gene sets, three genes (*SERPINB6*, *ANK1*, *OST4*) were in two optimal gene sets. Overlapped genes would be discussed in Section Discussion.

### Classification rules

**Figure 7:**
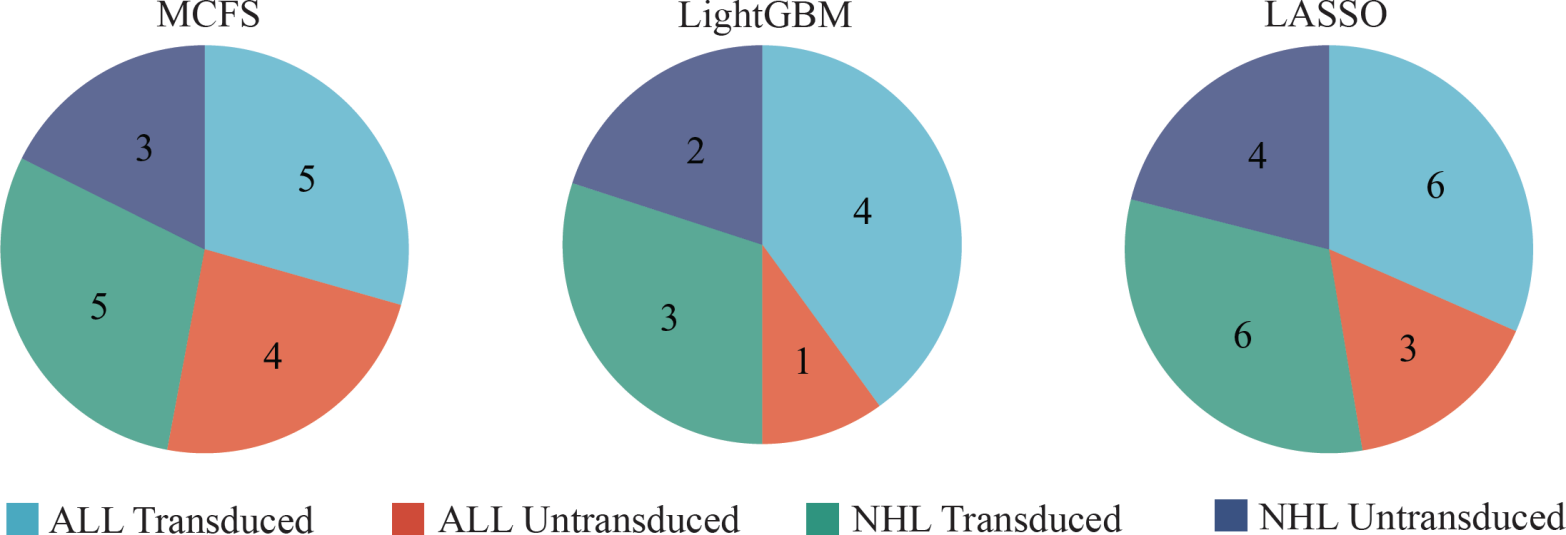
The number of rules extracted from the decision tree built on feature lists yielded by MCFS, LightGBM, and LASSO, respectively, on four classes.

Although the DT classifiers were generally weaker than RF classifiers, they can provide clues hidden in the investigated methylation profiles, which cannot be extracted by other classifiers. According to the IFS results on three feature lists, the best DT classifiers used top 591, 181 and 9 features in three lists, respectively. With these features, three DTs were learned on all samples. Each DT induced a rule set, which contained 17, 10 and 19 rules, respectively. Detailed rules are listed in **Table S4**. In each rule set, each class was assigned at least one rule, as shown in **Figure 7**. Following the rules in each rule set, we can determine the class of a test sample. Furthermore, their most contributions were the clear descriptions on different methylation patterns on CAR-transduced and untransduced T cells. This would be discussed in Section Discussion.

### Results of enrichment analysis

The optimal feature subsets for three feature ranking methods were determined by the IFS method. We mapped the methylation probes in three optimal feature subsets to genes, yielding a total of 341 genes. Then, the functional enrichment analysis was performed on these genes. The enrichment results are provided in **Table S5**. Two GO terms were enriched by 341 genes, whereas no KEGG pathways were enriched by these genes with FDR<0.05. GO enrichment result indicated that 12 of these genes were involved in the splicing process, suggesting that the transcripts of these genes may be involved in regulating CAR T-cell processes.

## Discussion

In this study, we applied several advanced machine learning algorithms to deeply mine the T-cell methylation profiles of patients with B-cell malignancies. Latent important genes were obtained and interesting classification rules were constructed. This section gave extensive analysis on these genes and rules.

### Top ranked genes in multiple algorithms

The first gene was *SERPINB6* (targeted by probes cg27001747 and cg04181408), which encoded a member of the serpin superfamily and ovalbumin-serpin subfamily (43, 44). *SERPINB6* appeared in both LightGBM and MCFS in the subset of optimal features. Both methylation probes were linked to the promoter and found in the 5ʹ-UTR region of *SERPINB6*, suggesting that they may affect the transcriptional regulation of this gene. Serpinb9, a homolog of SERPINB6, has been shown to protect T cells from Granzyme-B leaked from granules and also participates in T cell homeostasis (45, 46). Although the function of SERPINB6 in T cells has yet to be established, this protein is important to other immune cells. In neutrophils and monocytes, SERPINB6 inhibits Cathepsin G, thereby preventing programmed necrosis (47). Thus, SERPINB6 may play a role in the normal functioning of CAR-T cells. However, more research is needed to confirm this concept. Furthermore, SERPINB6 methylation has been linked to the risk of CLL pathogenicity (48). This result demonstrates the precision by which our method can identify CAR-T cell-specific genes and differential genes in B-cell malignancies.

The next probes identified were cg09405790 and cg02172579, which both targeted the gene body of *ANK1*. *ANK1* was found in the subset of optimum features in LightGBM and MCFS. ANK1 is a modular adaptor protein that mediates the connection of integral membrane proteins to the spectrin cytoskeleton (49). *ANK1* methylation has been shown to regulate the expression of microRNA-486-5p, which inhibits Interleukin-22 production by helper T cells *via* the Dock1/NF-B/Snail signaling pathway. Such process results in cancer suppression (50, 51).

The cg18756060 and cg04001935 probes were designed to detect the DNA methylation status in a specific intergenic region on chromosome 2 (chr2:27294139-27294915) according to GRCh37. Such region has been shown to be the coding region of gene *OST4*, and the protein encoded by *OST4* is an important subunit of oligosaccharyltransferase (OST). Similar to *SERPINB6* and *ANK1*, OST4 is an intersection feature of the optimal feature subsets of LightGBM and MCFS. Eukaryotic OSTs catalyze the N-glycosylation of nascent polypeptides in the lumen of the endoplasmic reticulum, a conserved biosynthetic process that diversifies the structure and function of proteins (52). Kumar et al. found that N-glycosylation activity remained elevated during the activation and expansion of human T cells, and lymphocytes in a resting state had lower N-glycosylation activity (53). These results suggest that OST was involved in T cell activation in transduced CARs, and that OST activity was influenced by methylation of *OST4*.

*PDCD5* (also known as *TFAR19*), which is targeted by the optimal features cg13563193, has been generally reported to participate in immunoregulation. *PDCD5* is at the top of the list of feature rankings obtained with the LASSO method. PDCD5 interacted with FOXP3 to promote FOXP3 acetylation, hence reducing effector cytokine production (54). Meanwhile, the methylation signal of PDCD5 was primarily found in the promoter region, which negatively regulated the PDCD5 expression and thus relaxed the immunosuppressive effect of Treg. This activity could explain the mechanism by which the CAR-T cells were activated and therefore appeared in our list. In addition, in hepatocellular carcinoma, the PDCD5 overexpression stimulates the promoter activity of KLF9, and the upregulation of KLF9 inhibits cell migration and proliferation (55). This phenomenon also suggests that the cg13563193 methylation signature may suppress the expression level of PDCD5. Yuan et al. have discovered that PDCD5 inhibits the production of proinflammatory mediators and promotes the secretion of anti-inflammatory cytokines by modifying the T-lymphocyte homeostasis (56). The two hallmark clinical toxicities associated with CAR-T cell therapy are cytokine release syndrome (CRS) and neurotoxicity (57, 58). The characteristics of CRS produce massive inflammation, suggesting a possible involvement of PDCD5 in this process.

The probe cg07632860 was developed to detect the methylation status of the transcription start site of the *DAPK2*. *DAPK2* is at the top of the list of feature rankings obtained with LASSO. *DAPK2* encodes a member of the serine/threonine protein kinase family, which functions as a tumor suppressor and regulates autophagic and apoptotic processes in various cell types (59, 60). When T lymphocytes are activated, they secrete inflammatory cytokines, such as TNF- and IL-6. During this process, DAPK2 is activated by T cell receptor, which inhibits T-cell activation (61, 62). We discovered that cg07632860 targeted the regulatory region of DAPK2, implying that it may limit the expression level of the protein. Meanwhile, DAPK2 expression has been found to be downregulated in ALL and NHL (63). Low levels of DAPK lead to T-cell activation, which implies the CAR-T cell activation mode. Furthermore, the inflammatory cytokines IL-17 and IL-32 have been demonstrated to use DAPK2 as a signaling mediator (64). Whether the production of cytokine storm, one of the side effects of CAR-T immunotherapy, is linked to DAPK2 is worthy of investigation.

The next predicted gene, *DNAJB6*, targeted by cg18753341, encodes a member of the DNAJ protein family, which is one of two key groups of molecular chaperones involved in biological activities, such as protein folding and oligomeric protein complex assembly. Strict control of the cell cycle process is essential for the proper functioning of T lymphocytes. Slfn1 has been shown to play an important role in the establishment and maintenance of T lymphocyte quiescence (65). Overexpression of DnaJB6 increases Slfn1 nuclear accumulation and causes cell-cycle arrest, whereas Slfn1 is mostly sequestered in the cytoplasm, and no cell-cycle arrest has been detected in *DnaJB6* knock-down cells (66). Furthermore, transgenic expression of DNAJB6 in T cells blocks Slfn1 degradation, enhances its nuclear import, and results in T cell proliferation suppression when T cell receptors are activated (66). In addition, DNAJB6 is neurotoxic when overexpressed in primary neurons, suggesting that it may be a potential locus for CAR-T treatment to eliminate side effects (67).

### Analysis of classification rules

In addition to the functional analysis of the top-ranked features, we also mined the specific rules used to distinguish each class based on the classification tree structure of the DTs. The rules of each class consisted of methylation probes and their signal intensities, and each methylation probe was linked to a gene to describe its function in greater depth.

The first rule was aimed to distinguish T cells derived from patients with ALL that have been transduced with CAR. *MYCN*, which is targeted by cg13799853, was an important site with low methylation, according to the classification rule based on LASSO results. In our classification rules, *MYCN* exhibited lower methylation levels. *MYCN* has been demonstrated to have lower methylation levels in relapsed children with B-cell acute lymphoblastic leukemia (B-ALL), which was consistent with the usage of *MYCN* in this study as a key feature to differentiate B-ALL (68). MYCN also downregulates DKK3 expression and activates the Wnt/β-catenin signaling pathway at the transcriptional level, boosting the development of B-ALL (69). Meanwhile, MYCN apparently decreases the interferon signaling, promoting a non-inflamed and T-cell infiltration-poor (“cool”) tumor microenvironment (70). In the classification rule based on the MCFS results, *HDGF* targeted by cg18593717 was an important locus, which exhibited a lower methylation level. HDGF has been demonstrated to cause Foxp3+ Treg differentiation and that Tregs decrease CD8+ cytotoxic T cell activity (71). This phenomenon suggests that *HDGF* may act as a potential gene driving the activation of CAR-T cell.

The second rule was used to distinguish the T cells derived from patients with ALL without transduced CARs. After constructing the DT by using the optimal subset obtained after MCFS, the classification rules were established. Among them, hypomethylation of the *ZBTB7A*, also known as LRF, was an important quantitative rule. Many studies have shown that ZBTB7A is closely associated with B and T cell differentiation and plays an important role in their fate decisions (72, 73). Meanwhile, dysregulation in B-cell maturation can lead to the development of autoimmune syndromes and B-cell malignancies (73). *ZBTB7* was described in the rules in our study, because it plays an important role in both immune processes and cancer development.

The next two rules were used to distinguish between CAR-transduced and untransduced T cells derived from patients with NHL. *TP73* targeted by cg10654015 appeared in our rules and exhibited a higher methylation status. *TP73* has been demonstrated to be frequently methylated in NHLs (74). This result is consistent with the highly methylated results of *TP73* found in our study, indicating the accuracy of our method. Furthermore, *TP73* deletion has been shown to impact lymphoma formation by several mechanisms, such as altered gene expression patterns, defective early T-cell growth, impaired apoptosis, and chromosomal abnormality accumulation (75). This phenomenon suggests that *TP73* may be a potential target for the modification of CAR-T cells.

## Conclusion

We applied a powerful computational strategy based on DNA methylation probe data to uncover the features of CAR T cells across diverse B-cell malignancies. The outcomes can be summarized in the three key components. First, a series of methylation signatures and genes were extracted, which can be used to distinguish cells from four different origins. The findings provided a theoretical foundation to precisely modify CAR T cells and treat B-cell malignancies. Second, efficient multi-class classifiers were built to aid in a more accurate delineation of T cells prior to treatment. The delineation of T cells facilitated the screening for T cells that could efficiently suppress cancer *in vivo* and further improve those that were not successfully transduced. Finally, some classification rules were built to specifically distinguish a particular class of cells. These rules aided to better understand the specific functions of CAR T cells by describing the degree of gene methylation.

## Data availability statement

Publicly available datasets were analyzed in this study. This data can be found here: https://www.ncbi.nlm.nih.gov/geo/query/acc.cgi?acc=GSE179414.

## Author contributions

TH and YD-C designed the study. LC and KYF performed the experiments. JS, FMH and FJ analyzed the results. JS, FMH and LC wrote the manuscript. All authors contributed to the research and reviewed the manuscript.

## Funding

This work was supported by the Strategic Priority Research Program of Chinese Academy of Sciences [XDB38050200, XDA26040304], National Key R&D Program of China [2018YFC0910403], the Fund of the Key Laboratory of Tissue Microenvironment and Tumor of Chinese Academy of Sciences [202002].

## Conflict of interest

The authors declare that the research was conducted in the absence of any commercial or financial relationships that could be construed as a potential conflict of interest.

## Publisher’s note

All claims expressed in this article are solely those of the authors and do not necessarily represent those of their affiliated organizations, or those of the publisher, the editors and the reviewers. Any product that may be evaluated in this article, or claim that may be made by its manufacturer, is not guaranteed or endorsed by the publisher.

## Supplementary material

Supplementary Table 1 Feature lists obtained by MCFS, LightGBM and LASSO.

Supplementary Table 2 Performance of IFS with different classification algorithms and feature lists.

Supplementary Table 3 Optimal gene sets based on MCFS, LightGBM, and LASSO methods, and their intersection results.

Supplementary Table 4 Classification rules generated by decision tree.

Supplementary Table 5 GO and KEGG enrichment results on the optimal gene sets of three feature ranking methods.
